# EGFR mutation frequency in Middle East and African non-small cell lung cancer patients: a systematic review and meta-analysis

**DOI:** 10.1186/s12885-018-4774-y

**Published:** 2018-09-14

**Authors:** Zineb Benbrahim, Teresita Antonia, Nawfel Mellas

**Affiliations:** 1 Department of Medical Oncology, Hassan II University Hospital, Faculty of Medicine and Pharmacy of Fez, University Sidi Mohammed Ben Abdellah, Fez, Morocco; 2 H. Lee Moffitt Cancer Center and Research Institute, Tampa, Florida USA

**Keywords:** Africa, EGFR mutation frequency, Middle East, Non-small cell lung cancer

## Abstract

**Background:**

Our goal was to investigate the prevalence of the epidermal growth factor receptor (EGFR) mutation in Middle East and African countries and to compare its prevalence with that shown in other populations.

**Methods:**

We used PubMed and the Cochrane Library databases to conduct a literature search using the terms “[EGFR] AND [mutation] AND [Non Small Cell Lung Cancer] AND [Middle East OR Africa].” We assessed studies published in English and French from 2004 until 2016.

**Results:**

Ten relevant studies were included in this systematic review. Overall, 1215 patients with non-small cell lung cancer (NSCLC) were included in this analysis. The overall ratio of male to female patients was 2.15. Of total patients included, 41.1% had never smoked and 85.8% had been diagnosed with adenocarcinoma. In 8 of the 10 studies, polymerase chain reaction (PCR) analyses were conducted to identify EGFR mutations. In total, 257 patients had an *EGFR* mutation, corresponding to a prevalence of 21.2%. The most frequent abnormality detected in all of the studies was in exon 19. In addition, all studies concluded the presence of a correlation between EGFR mutation status and female sex, non-smoking status, and adenocarcinoma subtype.

**Conclusions:**

The EGFR mutation frequency in Middle East and African patients is higher than that shown in white populations but still lower than the frequency reported in Asian populations.

## Background

Lung cancer is a worldwide public health issue. According to updated statistics from the International Agency for Research on Cancer, lung cancer is the most frequent and the most common cause of cancer mortality in men [1]. Non-small cell lung cancer (NSCLC) represents 80% of overall lung cancers [1].

Despite therapeutic improvements over the past decade, survival rates remain low [2]. Recent advances in our understanding of signaling pathways suggest that epidermal growth factor receptor (EGFR) mutations are potential therapeutic targets leading to improved response and survival with specific inhibitors in patients carrying such mutations [3].

EGFR exists on the cell surface and is activated by binding of its specific ligands. Somatic mutations of the *EGFR* gene lead to the constant activation of the receptor, which causes uncontrolled cell division [4].

These mutations are more common in patients with adenocarcinomas, in women, and in non-smokers [5]. Some studies have demonstrated a genetic divergence of EGFR mutation rates according to ethnicity [6, 7]. The highest frequency is found in Asian populations (47%) and the lowest occurs in those with Oceanian ethnicity (12%). The frequency of EGFR mutations in Middle East and African countries has not been determined.

In this study, we conducted a systematic review of publications related to the frequency of the EGFR mutation in Middle East and African regions to describe the prevalence of the EGFR mutation in this region and compare the results with other populations.

## Methods

We used PubMed and the Cochrane Library databases to identify published studies of EGFR mutation frequency in NSCLC in the Middle East and Africa. The following search terms were used: [EGFR] AND [mutation] AND [Non Small Cell Lung Cancer] AND [Africa OR Middle East]. We excluded the following terms: [Review] AND [Editorial] AND [Letter]. We then examined the reference lists of these publications for additional relevant studies not identified in the search. We also searched abstracts from conference proceedings of the American Society of Clinical Oncology (ASCO), the European Society for Medical Oncology (ESMO), and the World Lung Cancer Conference (WLCC) to identify unpublished studies. We used both English and French languages for the search. We assessed studies published from January 1, 2004 until July 1, 2016.

From this initial research, we reviewed publications to be sure that studies with small numbers of patients were included as published data are scarce in Middle East and African populations. An additional literature search that included specific country names yielded relevant publications for these specific populations. The study results were then synthesized.

## Results

### Literature research

The initial literature research of original articles in PubMed and the Cochrane Library yielded 16 publications; of these, 5 articles containing relevant EGFR mutation frequency data were selected. This first literature research provided data from Lebanon, Saudi Arabia, and Turkey. The additional literature search based on the same criteria with specific names of countries (Egypt, Syria, Jordan, Iraq, Iran, Kuwait, Bahrain, Yemen, Palestine, Oman, United Arab Emirates, and Qatar from the Middle East and 51 countries from Africa) yielded 20 additional articles of which 10 were relevant.

**Fig. 1:**
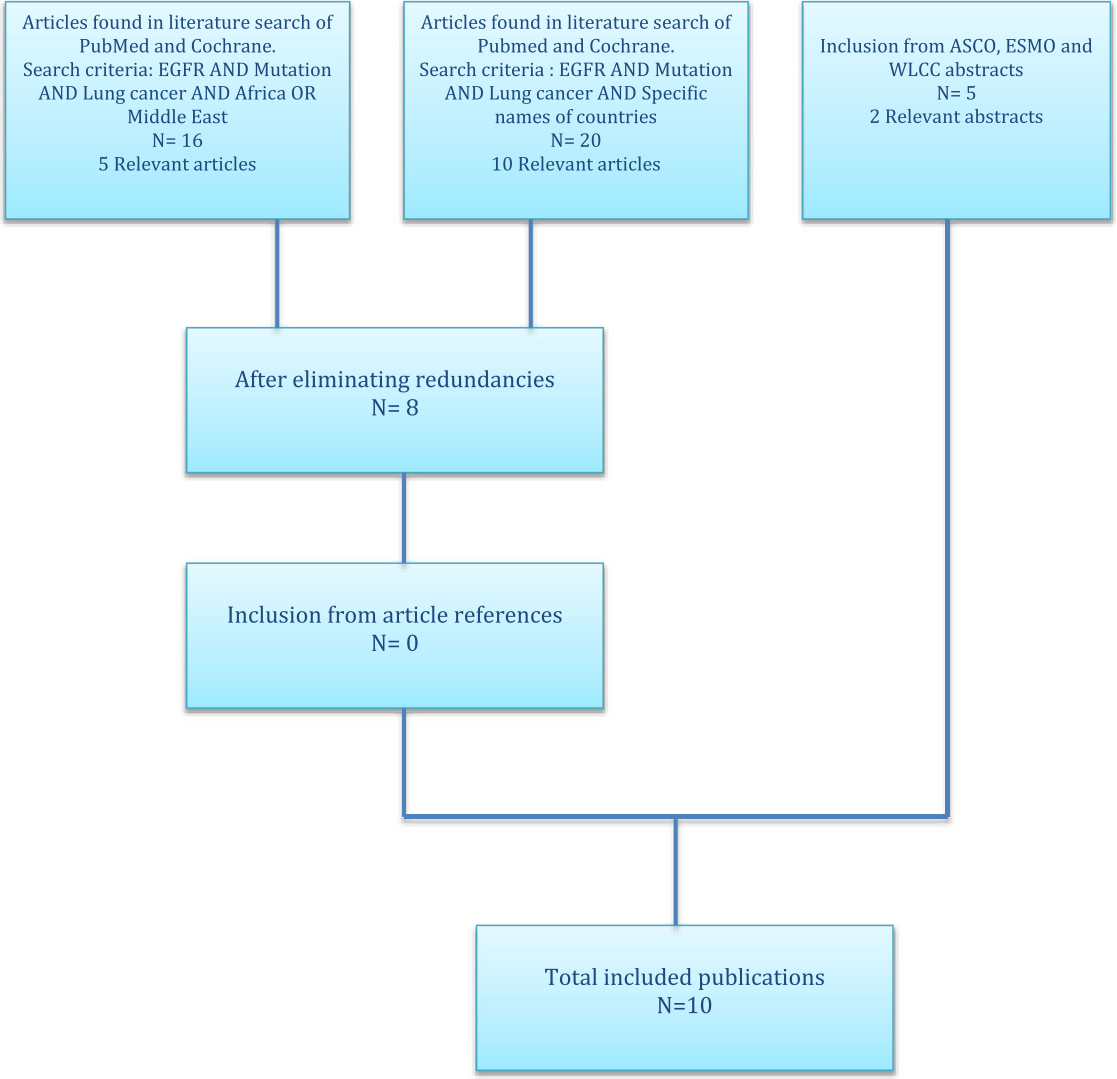
Flow of the studies identified and included in this systematic review

After redundancies were eliminated, 8 articles were identified. A reference list examination did not reveal any additional articles. Two additional unpublished studies were identified while assessing abstracts from ASCO, ESMO, and WLCC. This resulted in a total of 10 publications included in this review (Fig. 1).

### Description of study design

Nine of the 10 studies (90%) were cross-sectional; for the remaining study (published in the ASCO abstract book), the study design was not clear. Six of these cross-sectional studies (66.7%) were performed among non-consecutive patients and consequently have a level of evidence III according to the Oxford Centre for Evidence-based Medicine 2011 Levels of Evidence [8]. The remained 3 studies were performed among consecutive patients and therefore have a level II according to the same classification. Six studies (60%) were single-center studies, and 4 (40%) were multicenter studies.

### Description of sample sizes and included regions

Forty percent of studies included less than 100 patients, reducing the power of these studies to estimate the true frequency of the EGFR mutation. The median number of included patients was 121, and the number of patients ranged from 25 (Turkish study) to 230 (Gulf region study).

Most of the included studies (8/10, 80%) reported results from the Middle East; the remaining studies were from North Africa. No data were found from Central and South Africa. Inclusion and exclusion criteria were well defined in only two studies.

### Biases and confounding patient characteristics

Age, sex, and histological subtypes of included patients were reported in all of the studies; however, data on smoking status were only available in 9 studies whereas data on ethnical background were precised in an exclusive study reported in the Gulf region [16]. These characteristics were summarized using means and standard deviations for the ordinal variables and frequencies for the categorical ones.

Male patients were predominant in all of the studies; the overall ratio of males to females was 2.15 (Table 1). The smoking status distribution was heterogeneous between the studies, with the frequency of never-smokers varying from 10.4% [13] to 80.9% [15]. In the 9 studies that reported smoking status, approximately 41.1% of patients had never smoked (Table 2). We determined that 85.8% of the patients included in the studies had an adenocarcinoma; however, two studies [10, 18] only reported on patients with this subtype (Table 3).

**Table 1:** Male-female distribution of patients with non-small cell lung cancer

Country	Author	Male, No (%)	Female, No. (%)
Overall		829 (68.2%)	386 (31.8%)
Lebanon	Naderi et al. [9]	123 (61.2%)	78 (38.8%)
	Fakhruddin et al. [10]	72 (67.9%)	34 (32.1%)
	Kattan et al. [11]	102 (59.8%)	68 (40.2%)
Egypt	Zaki et al. [12]	40 (80%)	10 (20%)
Turkey	Unal et al. [13]	41 (85.4%)	7 (14.6%)
	Bircan et al. [14]	21 (84%)	4 (16%)
Saudi Arabia (SA)	Al-Kuraya et al. [15]	37 (78.7%)	10 (21.2%)
SA-UAE-Qatar	Jazieh et al. [16]	162 (70.4%)	68 (29.6%)
SA-UAE-Egypt	Jazieh et al. [17]	140 (69.7%)	61 (30.3%)
Morocco	Errihani et al. [18]	91 (66.4%)	46 (33.6%)

**Table 2:** Distribution of non-small cell lung cancer patients by smoking status

Country	Author	Never-smokers, NO. (%)	Ever-smokers, No.(%)
Overall		383 (41.1%)	550 (58.9%)
Lebanon	Naderi et al. [9]	44 (21.9%)	157 (78.1%)
	Fakhruddin et al. [10]	59 (76.6%)	18 (23.4%)
	Kattan et al. [11]	39 (22.9%)	131 (77.1%)
Egypt	Zaki et al. [12]	No data	No data
Turkey	Unal et al. [13]	5 (10.4%)	43 (89.6%)
	Bircan et al. [14]	6 (26.1%)	17 (73.9%)
Saudi Arabia (SA)	Al-Kuraya et al. [15]	38 (80.9%)	9 (19.1%)
SA-UAE-Qatar	Jazieh et al. [16]	134 (58.3%)	96 (41.7%)
SA-UAE-Egypt	Jazieh et al. [17]	No data	No data
Morocco	Errihani et al. [18]	58 (42.3%)	79 (57.7%)

**Table 3:** Distribution of non-small cell lung cancer patients by histological subtype

Country	Author	Adenocarcinoma, No. (%)	Other subtypes No. (%)
Overall		1043 (85.8%)	172 (14.2%)
Lebanon	Naderi et al. [9]	182 (90.5%)	19 (9.5%)
	Fakhruddin et al. [10]	106 (100%)	0 (0%)
	Kattan et al. [11]	157 (92.4%)	13 (7.6%)
Egypt	Zaki et al. [12]	23 (46%)	27 (54%)
Turkey	Unal et al. [13]	32 (66.7%)	16 (33.3%)
	Bircan et al. [14]	14 (56%)	11 (44%)
Saudi Arabia (SA)	Al-Kuraya et al. [15]	26 (55.3%)	21 (44.7%)
SA-UAE-Qatar	Jazieh et al. [16]	191 (83.0%)	39 (17.0%)
SA-UAE-Egypt	Jazieh et al. [17]	175 (87.1%)	26 (12.9%)
Morocco	Errihani et al. [18]	137 (100%)	0 (0%)

### Methods of EGFR mutation research

We assessed homogeneity of studies included in the review regarding methods of detection of the EGFR mutation. We concluded that all samples had been formalin-fixed, paraffin-embedded. In 9 articles (90%), the EGFR mutation research was performed using polymerase chain reaction (PCR) analyses. The PCR kits enabled detection of mutations in exons 18 to 21 of the EGFR gene.

#### EGFR mutation frequency

Overall, 1215 patients with NSCLC were included in this analysis. Of these, 257 patients had an *EGFR* mutation, corresponding to a prevalence of 21.2%. This frequency varied from 44% in Turkey [14] to 2.1% in Saudi Arabia [15]. These data are summarized in Table 4. The most frequent abnormality detected in all of the studies was in the exon 19 (Table 5), which is correlated with the best response to EGFR inhibitors.

**Table 4:** Frequency of EGFR mutation in non-small cell lung cancer patients by country

Country	Author	Number of patients	Frequency of EGFR mutation, No. (%)
Overall		1215	257 (21.2%)
Lebanon	Naderi et al. [9]	201	24 (11.9%)
	Fakhruddin et al. [10]	106	9 (8.5%)
	Kattan et al. [11]	170	22 (12.9%)
Egypt	Zaki et al. [12]	50	11 (22%)
Turkey	Unal et al. [13]	48	18 (37.5%)
	Bircan et al. [14]	25	11 (44%)
Saudi Arabia (SA)	Al-Kuraya et al. [15]	47	1 (2.1%)
SA-UAE-Qatar	Jazieh et al. [16]	230	66 (28.7%)
SA-UAE-Egypt	Jazieh et al. [17]	201	66 (32.8%)
Morocco	Errihani et al. [18]	137	29 (21.2%)

**Table 5:** Distribution of EGFR Mutations in Non-Small Cell Lung Cancer Patients by mutation types

Country	Author	G719X mutation in exon 18 N (%)	Deletion on exon 19 N (%)	Insertion in exon 20 N (%)	L858R mutation on exon 21 N (%)
Overall		15	151	13	80
Lebanon	Naderi et al. [9]	1 (4.2%)	12 (50.0%)	1^a^ (4.2%)	10 (41.6%)
	Fakhruddin et al. [10]	0 (0%)	8 (88.9%)	0 (0%)	1 (11.1%)
	Kattan et al. [11]	1 (4.5%)	11 (50%)	1 (4.5%)	9 (41.0%)
Egypt	Zaki et al. [12]	0 (0%)	11 (100%)	0 (0%)	0 (0%)
Turkey	Unal et al. [13]	0 (0%)	7 (38.9%)	9 (50%)	2 (11.1%)
	Bircan et al. [14]	0 (0%)	7^a^ (63.6%)	0 (0%)	4^a^ (36.4%)
Saudi Arabia (SA)	Al-Kuraya et al. [15]	0 (0%)	0 (0%)	1 (100%)	0 (0%)
SA-UAE-Qatar	Jazieh et al. [16]	4^a^ (6.1%)	36 (54.5%)	0 (0%)	26 (39.4%)
SA-UAE-Egypt	Jazieh et al. [17]	7 (10.3%)	38^a^ (57.4%)	0 (0%)	21^a^ (32.3%)
Morocco	Errihani et al. [18]	2 (6.9%)	20 (68.95%)	1 (3.45%)	6 (20.7%)

^a^6 cases in the reviewed population present double mutations mostly in exons 19 and 21

#### Correlation of the EGFR mutation with predictive factors

All 10 studies reported a higher frequency of EGFR mutations in female versus male patients. Eight studies compared EGFR mutation frequencies versus smoking status, with all reporting a higher frequency of EGFR mutations in non-smokers than in ever-smokers. In 5 of the 6 studies reporting EGFR mutation status according to the histological subtype, there was a higher frequency of EGFR mutation in adenocarcinomas compared with other subtypes: (13 vs 0% [9], 40.6 vs 31.3% [13], 25 vs 0% [15], 32.46 vs 10.25% [16] and 36.25 vs 15,38% [17]).

Finally, in the one study reporting EGFR mutation according to the ethnical background, there was no significant difference between Arabic and non Arabic patients [16].

In our correlative analysis of all cases, the prevalence of the EGFR mutation was higher in female versus male patients (OR = 1.87; 95% CI, 1.44–2.42) and in non-smokers versus ever-smokers (OR = 8.81; 95% CI, 6.42–12.10). Regarding the association between having the EGFR mutation and adenocarcinoma versus other histological subtypes, we determined an odds ratio of 0.73 in adenocarcinomas versus other subtypes (95% CI, 0.47–1.12). However, correlations between the EGFR mutation status and histological subtypes were not statistically significant (Table 6).

**Table 6:** Correlation between egfr mutation status and sex, smoking status, and histological subtypes in non-small cell lung cancer patients included in the review

Overall patients	EGFR mutated	EGFR wild-type	Odds Ratio (95% confidence interval)
Sex			
Female	156	210	1.87 (1.44-2.42)
Male	214	538	
Smoking status			
Never smokers	203	102	8.81 (6.42-12.10)
Ever smokers	107	474	
Histology			
Adenocarcinomas	222	200	0.73 (0.47-1.12)
Other subtypes	64	42	

## Discussion

This systematic review revealed that the EGFR mutation is more frequent in Middle East and African population than in white populations but still lower than frequencies reported in Asian populations. This prevalence was expected to be different based on the different geographic location and the racial and ethnic backgrounds of this population.

The most frequent abnormality detected in all of the studies was in exon 19. This was consistent with data from the literature where the average frequency of the exon 19 mutation approaches 40% among all EGFR mutations [19].

The EGFR mutation status was correlated with both female sex and non-smoking status, in accordance with known data in the literature [5]. However, no significant correlation was found with histological subtype in our study, which may be due to the low number of non-adenocarcinoma patients included in the studies reviewed (14.2% of overall patients).

Results of this systematic review should be considered cautiously due to several limitations. First, 4 of the 10 studies included less than 50 patients; the statistically low power of these studies reduces the chance to detect the true prevalence of the EGFR mutation in our studied populations. Second, differences in male/female, smoking status, and adenocarcinoma proportions and limited data about stage are potential sources of heterogeneity among the included studies. In addition, the cross-sectional nature of the studies, especially the inclusion of non-consecutive patients, may have been associated with a selection bias. For example, patients may have been included by their treating physicians on the basis of their demographic and clinical characteristics. This hypothesis was confirmed when reviewing demographic data of lung cancer in the Middle East, which showed lower proportion of the female gender in the epidemiological data comparatively to their proportion among patients included in our reviewed studies (20.3% in the epidemiological data (20) versus 31.76% in our reviewed studies). Furthermore, the cases included in the studies that we reviewed were mostly adenocarcinomas (85.8%), which is not consistent with data from the Middle East region where squamous carcinomas are predominant [20]. Third, the methods used to determine EGFR mutation status lacked homogeneity among the included studies; in some studies, the mutations were confirmed by sequencing whereas in others they were not. Finally, publication bias may have been present in this analysis since we included only English and French language publications.

## Conclusions

The EGFR mutation frequency in Middle East and Africa populations is higher than in white populations but still lower than those reported in Asian populations. The results of this systematic review should be considered cautiously given the design of included studies (mainly non-consecutive cross-sectional), the characteristics of included patients, and the heterogeneity of the EGFR mutation research methods. Further prospective multicenter studies with standardized inclusion and exclusion criteria and research methods are required to more definitively evaluate patients from the Middle East and Africa with NSCLC for this mutation and therefore their eligibility to receive EGFR inhibitor therapies in this region. In the meantime, EGFR mutation status should be analyzed for all patients with NSCLC in the Middle East and Africa as per the National Comprehensive Cancer Network guidelines.

## Abbreviations

ASCO: American Society of Clinical Oncology
EGFR: Epidermal growth factor receptor
ESMO: European Society for Medical Oncology
NSCLC: Non-small cell lung cancer
OR: Odds Ratio
PCR: Polymerase chain reaction
WLCC: World Lung Cancer Conference
